# Checkpoint Regulation of Nuclear Tos4 Defines S Phase Arrest in Fission Yeast

**DOI:** 10.1534/g3.119.400726

**Published:** 2019-11-12

**Authors:** Seong M. Kim, Vishnu P. Tripathi, Kuo-Fang Shen, Susan L. Forsburg

**Affiliations:** Program in Molecular and Computational Biology, University of Southern California, Los Angeles CA 90089

**Keywords:** Checkpoint, Cell cycle, Tos4, fission yeast, S phase

## Abstract

From yeast to humans, the cell cycle is tightly controlled by regulatory networks that regulate cell proliferation and can be monitored by dynamic visual markers in living cells. We have observed S phase progression by monitoring nuclear accumulation of the FHA-containing DNA binding protein Tos4, which is expressed in the G1/S phase transition. We use Tos4 localization to distinguish three classes of DNA replication mutants: those that arrest with an apparent 1C DNA content and accumulate Tos4 at the restrictive temperature; those that arrest with an apparent 2C DNA content, that do not accumulate Tos4; and those that proceed into mitosis despite a 1C DNA content, again without Tos4 accumulation. Our data indicate that Tos4 localization in these conditions is responsive to checkpoint kinases, with activation of the Cds1 checkpoint kinase promoting Tos4 retention in the nucleus, and activation of the Chk1 damage checkpoint promoting its turnover. Tos4 localization therefore allows us to monitor checkpoint-dependent activation that responds to replication failure in early *vs.* late S phase.

The cell cycle proceeds through a rhythmic pattern of oscillators driven by cell-cycle specific transcription, patterns of protein modification, and protein degradation (reviewed in (Bertoli *et al.* 2013; Malumbres 2014; Alber *et al.* 2019)). Fission yeast is an important model system for studying cell cycle dynamics and genome stability. The rod-shaped cells are divided by medial fission with distinct cell morphologies (Piel and Tran 2009). Typically, mitosis is completed and S phase begins when cells are in a binucleate stage, prior to septation (Gomez and Forsburg 2004; Peng *et al.* 2005; Piel and Tran 2009). Thus, new-born cells are considered to be in late S to G2 phase, while S phase begins in binucleates (MacNeill and Fantes 1997). Distinguishing early from late S phase is typically done by monitoring nuclear DNA content by methods such as FACS or BrdU (Hodson *et al.* 2003; Sabatinos and Forsburg 2015b). Isotopic labeling methods suggest that the bulk of DNA synthesis is complete in a short time, leading to the conclusion that S phase is quite short and G2 phase extended (Nasmyth *et al.* 1979).

However, many replication mutants in fission yeast show an approximately 2C DNA content upon cell cycle arrest; based on genetic studies, this has been proposed to be late S phase (*e.g.*, (Nurse *et al.* 1976; Nasmyth and Nurse 1981; Coxon *et al.* 1992; Forsburg and Nurse 1994)). Whether this arrest represents failure to duplicate specific late regions remains to be seen. Generally, late-replicating genome regions show increased prevalence of mutations and fragile sites (Le Beau *et al.* 1998; Stamatoyannopoulos *et al.* 2009; Lang and Murray 2011). Very late DNA replication has been observed, even into M phase for repair synthesis (Widrow *et al.* 1998; Bergoglio *et al.* 2013; Minocherhomji *et al.* 2015). Indeed, models of replication stress increasingly suggest the issue is not within early S phase but disruptions of chromosome segregation during mitosis (Zeman and Cimprich 2014; Minocherhomji *et al.* 2015; Zhang *et al.* 2019).

We are interested in identifying early S phase cells and distinguishing them from late S phase or G2. Recent advances in live cell imaging have been accompanied by developing markers that are specific to particular cell cycle compartments. For example, the FUCCI (Fluorescent Ubiquitination-based Cell Cycle Indicator) system has been deployed using tagged, ubiquitylated proteins that are specific to G1/S or G2 cells (Sakaue-Sawano *et al.* 2008) and further refined by additional markers specific to G0 (Oki *et al.* 2014) or to multiple cell cycle phases (Bajar *et al.* 2016). These proteins vary temporally and spatially, giving a snapshot of cells in a particular cell cycle phase. There are excellent markers for mitotic landmarks including fluorescently tagged spindle pole body protein Sad1 (King and Drivas 2008) or tubulin (Sawin and Tran 2006), and septation is easily examined under light microscopy (Minet *et al.* 1979). We have developed and employed numerous tools to identify and characterize features of DNA synthesis and replication stress, including fluorescent tagged RPA and Rad52 proteins (Sabatinos *et al.* 2012, 2015; Green *et al.* 2015; Sabatinos and Forsburg 2015a; Escorcia and Forsburg 2017), and have also examined abnormal mitotic divisions in response to replication stress including nuclear envelope, cell membrane, and histone markers (Sabatinos *et al.* 2012, 2015; Escorcia and Forsburg 2017).

The forkhead-associated domain (FHA)-containing DNA binding protein Tos4 is conserved in budding and fission yeast (Kiang *et al.* 2009; Bastos de Oliveira *et al.* 2012; Smolka *et al.* 2012). It is known to be regulated by the G1/S phase master transcription factor MBF (MluI-binding factor transcriptional complex) in both species (Kiang *et al.* 2009; Bastos de Oliveira *et al.* 2012). In fission yeast, Tos4 is a non-essential protein with relatively minor phenotypes (Kim *et al.*, 2010; Hayles *et al*. 2013) which is phosophorylated during the G1/S phase (Swaffer *et al*., 2016). Overproduction leads to cell cycle delay (Vachon *et al*., 2013) . Of particularly interest to us, Tos4-GFP shows periodic accumulation in the nucleus coincident with S phase, consistent with its known regulation and the maturation timing of GFP (Kiang *et al.* 2009; Bastos de Oliveira *et al.* 2012; Escorcia *et al.* 2019; Shen and Forsburg 2019). In fission yeast, this has been exploited in studies of cyclical re-replication induced by cyclin inhibition (Kiang *et al.* 2010).

In this study, we characterize Tos4-GFP as a dynamic marker for S phase and determine its response to a variety of replication stresses, using both fluorescence microscopy and flow cytometry. We observe consistent timing of Tos4 accumulation relative to SPB duplication and septation in wild type cells. Tos4 persists in the nucleus of cells arrested in S phase by hydroxyurea (HU) or cell cycle mutant *cdc22-M45*, treatments which activate the replication checkpoint kinase Cds1. Consistent with this, accumulation of nuclear Tos4 requires Cds1, kinase activity, and the FHA domain. Surprisingly, however, replication mutants that show presumed late S phase arrest lack nuclear Tos4. This suggests that Tos4 specifically delineates an early stage of S phase and leads to the possibility that “late S phase” defined by replication mutants overlaps with what we commonly call G2 phase in which low yet detectable levels of DNA synthesis is occurring (Kelly and Callegari 2019).

## Materials and Methods

### Yeast strains and media

*S. pombe* strains (Table 1) were grown in supplemented Edinburgh minimal medium (EMM) for live cell imaging, Western blot, and flow cytometry. Cells were treated with12 mM hydroxyurea (HU, Sigma), incubated at 36° for 4h, or pre-treated with 12 mM HU for 2 h at 25° and then incubated at 36° for 4h.

**Table 1:** Yeast strains used in this study

Strain	Genotype	Source
FY8222	*h- Tos4-GFP*::*KanMX6 Sad1+*::*DsRed-LEU2 leu1-32 ura4-D18 ade6-M210 can1-1*	Our stock
FY8678	*h- cdc21-M68 Tos4-GFP*::*KanMX6 Sad1+*::*DsRed-LEU2 ura4-D18 leu1-32 ade6-(M216 or 704)*	Our stock
FY8682	*h+ mcm4 (cdc21-m68)-ts-dg*::*ura4+ Tos4-GFP*::*KanMX6 Sad1+*::*DsRed-LEU2 ura4-D18 leu1-32*	Our stock
FY8851	*h+ cdc25-22 Tos4-GFP*::*KanMX6 leu1-32 ura4-D18 ade6-M210*	Our stock
FY8853	*h+ cdc10-V50 Tos4-GFP*::*KanMX6 Sad1+*::*DsRed-LEU2 ura4-D18 leu1-32*	Our stock
FY8855	*h- cdc22-M45 Tos4-GFP*::*KanMX6 Sad1+:DsRed-LEU2 cdc22-M45 ura4-D18 ade6-M210 leu1-32*	Our stock
FY8939	*h- nda3-KM311 Tos4-GFP*::*KanMX6 leu1-32 ura4-D18*	Our stock
FY9062	*h- ∆cds1*::*ura4+ Tos4-GFP*::*KanMX6 Sad1+*::*DsRed-LEU2 ura4-D18 leu1-32*	Our stock
FY9064	*h- cdc21-c106*::*HphMx Tos4-GFP*::*KanMX6 Sad1+*::*DsRed-LEU2 ura4-D18 ade6-M210 leu1-32*	Our stock
FY9074	*h+ cdc17-M75(kg) Tos4-GFP*::*KanMX6 Sad1+*::*DsRed-LEU2 ura4-D18 leu1-32 can1-1*	Our stock
FY9075	*h- cdc17-K42(kg) Tos4-GFP*::*KanMX6 Sad1+*::*DsRed-LEU2 ura4-D18 leu1-32 ade6-M210 can1-1*	Our stock
FY9120	*h- cut9-665 Tos4-GFP*::*KanMX6 sad1+*::*DsRed-LEU2 ura4-D18 leu1-32 ade6-M210*	Our stock
FY9126	*h- ∆cds1*::*ura4+ cdc21-M68 Tos4-GFP*::*KanMX6 Sad1+*::*DsRed-LEU2 ura4-D18 leu1-32*	Our stock
FY9127	*h+ ∆chk1*::*ura4+ cdc21-M68(mcm4)-dg*::*ura4+ Tos4-GFP*::*KanMX6 Sad1+*::*DsRed-LEU2 ura4-D18 leu1-32*	Our stock
FY9128	*h+ cdc18 -K46 Tos4-GFP*::*KanMX6 Sad1+*::*DsRed-LEU2 leu1-32 ura4-D18 ade6-M210*	Our stock
FY9129	*h- ∆cds1*::*ura4+ cdc22-M45 Tos4-GFP*::*KanMX6 Sad1+*::*DsRed-LEU2 ura4-D18 leu1-32*	Our stock
FY9131	*h- cdc6-23 Tos4-GFP*::*KanMX6 Sad1+*::*DsRed-LEU2 ura4-D18 leu1-32*	Our stock
FY9133	*h- pold-ts2 (cdc6-ts2) Tos4-GFP*::*KanMX6 Sad1+*::*DsRed-LEU2 ura4-D18 leu1-32*	Our stock
FY9135	*h+ cdc27-K3 Tos4-GFP*::*KanMX6 Sad1+*::*DsRed-LEU2 ura4-D18 leu1-32 can1-1*	Our stock
FY9157	*h- rad4-116 Tos4-GFP*::*KanMX6 Sad1+*::*DsRed-LEU2 ura4-D18 leu1-32 ade6-M210 can1-1*	Our stock
FY9158	*h+ hsk1-1312 Tos4-GFP*::*KanMX6 Sad1+*::*DsRed-LEU2 ura4-D18 leu1-32 ade6-M210 his3-D1*	Our stock
FY9159	h+ cds1-FHA*:2HA6His:ura4+:leu1+ Tos4-GFP::*KanMX6 leu1-32 ura4-D18*	P. Russell
FY9180	*h- goa1-U53 (sna41ts) Tos4-GFP*::*KanMX6 Sad1+*::*DsRed-LEU2 leu1-32 ura4-D18 his3-D1 ade6-M210*	Our stock
FY9283	*h+ cut4-533 Tos4-GFP*::*KanMX6 Sad1+*::*DsRed-LEU2 leu1-32*	Our stock
FY9284	*h+ nuc2-663 Tos4-GFP*::*KanMX6 Sad1+*::*DsRed-LEU2 leu1-32 ura4-D18*	Our stock

### Live-cell microscopy

Cells cultured in supplemented EMM media were placed on 2% agarose pads sealed with VaLaP (1/1/1 [wt/wt/wt] Vaseline/lanolin/paraffin) for live-cell imaging. Images were acquired using a DeltaVision microscope (with softWoRx version 4.1; GE, Issaquah, WA) using a 60x (NA 1.4 PlanApo) lens, solid-state illuminator, and 12-bit CCD camera. Images were deconvolved and maximum intensity projected for fluorescence images (sofrWoRX) and transmitted light images were inverted and added for outline of the cells (ImageJ) (Schindelin *et al.* 2012).

### Western blot

Proteins extracts were prepared from equal number of Tos4-GFP cells in asynchronous culture grown in supplemented EMM media, after treatment with 12 mM hydroxyurea (HU), and after washing twice with media for release from HU. Cells in mid-log phase were harvested and whole-cell protein extract was prepared by vortexing acid-washed glass beads in 20% trichloroacetic acid (TCA) and washing beads with 5% TCA. Lysates were boiled for 5 min in Laemmli Sample buffer (4%SDS, 60 mM Tris-HCl, pH 6.8, 5% glycerol, 4% 2-mercaptoethanol, 0.01% bromophenol blue) and analyzed by 4–12% SDS-PAGE (Expedeon), followed by immunoblotting with rabbit anti-GFP (Abcam 290; 1:1000) and rabbit anti-cdc2 (gift from Nurse lab; 1:4000) as loading control. After secondary antibody (anti-rabbit Alexa Flour 488; 1:4000) incubation, blots were developed using Amersham Typhoon biomolecular imager.

### Flow cytometry

Cells were fixed in cold 70% ethanol and processed in 50 mM sodium citrate, 100 μg/ml RNase A, and 8 µg/ml propidium iodide (PI). Samples were sonicated and then run on the flow cytometer (BD Accuri C6 Plus) for GFP and PI.

### Statistical Analysis

A two-tailed Student’s *t*-test was used to determine significance: * *P* < 0.05, ** *P* < 0.01, *** *P* < 0.001, n.s. not significant. Error bars represent Standard Error (SE).

### Data availability

Strains and plasmids are available upon request. The authors affirm that all data necessary for confirming the conclusions of the article are present within the article, figures, and tables. Supplemental material available at figshare: https://doi.org/10.25387/g3.10257524.

## Results

### Dynamics of Tos4 in cycling wild type cells

**Figure 1:**
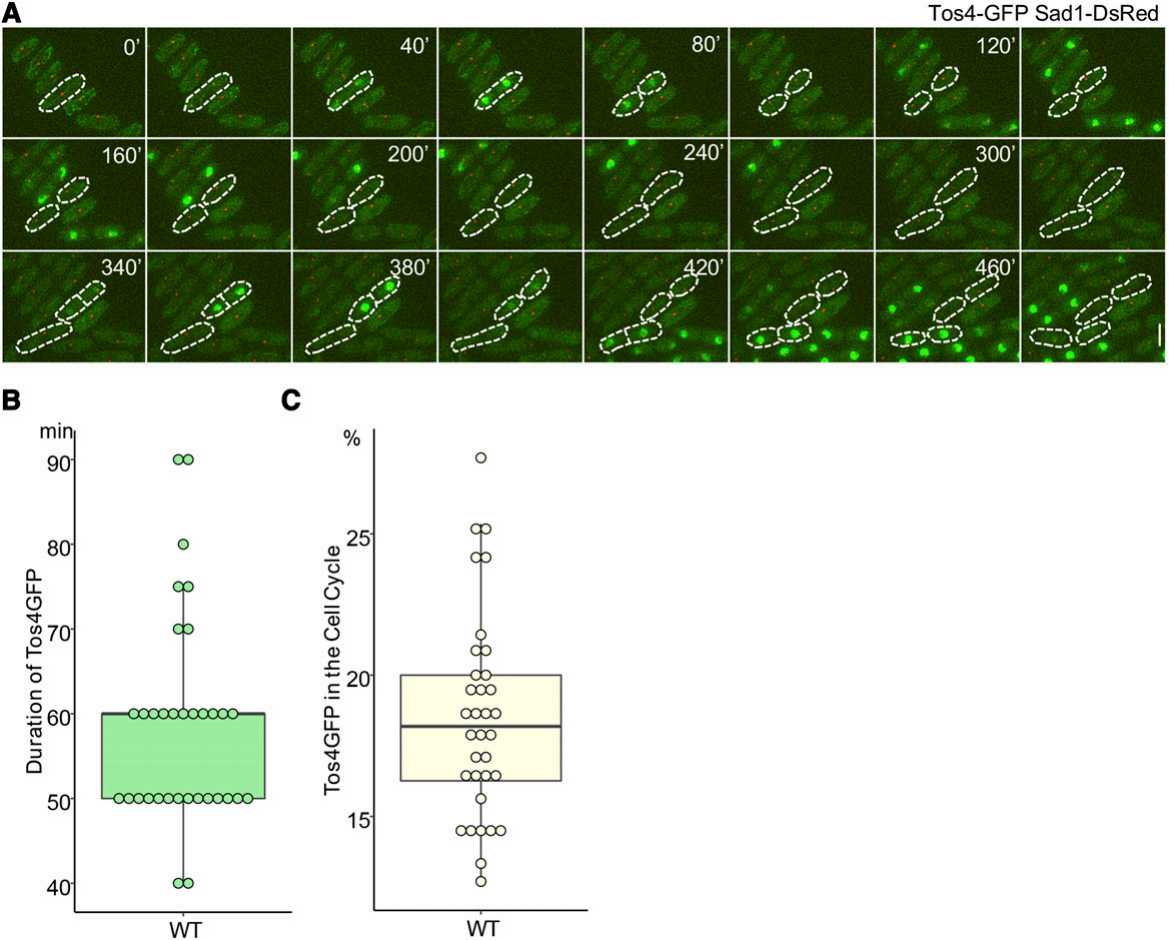
Tos4-GFP is present in nuclei of dividing cells. (A) Live cell imaging of WT cells containing Tos4-GFP and Sad1-DsRed was followed at 25°C for 8 hr. Nuclear Tos4-GFP signal is observed after duplication of SPBs and it disappears from the nucleus before cells enter the next round of mitosis. Scale bar, 5 µm. (B) Duration of the presence of nuclear Tos4-GFP. The presence of nuclear Tos4-GFP was determined by measuring the nuclear Tos4-GFP signals using the ImageJ software (Schindelin *et al.* 2012). Nuclear Tos4-GFP signals was counted as a positive when nuclear Tos4-GFP signal is greater than 50 (scale 0-255) after the background subtraction using ImageJ. (C) Ratio of nuclear Tos4-GFP duration *vs.* the cell cycle duration is presented. Duration of the cell cycle was determined by measurement of the timing between the first and second separation of SPBs. Sad1-DsRed was used to follow the separation of SPBs.

To observe the dynamics of Tos4 accumulation in relation to the cell cycle, we imaged cells with Tos4-GFP and the spindle pole marker Sad1-DsRed. Tos4 accumulated in the nucleus of dividing cells, correlating to cells early S phase (Figure 1A and Supplemental Material, Movie 1). Nuclear Tos4-GFP was present largely in binucleate cells, corresponding to early S phase, and was also observed in some short mononucleate cells (newborns) following completion of septation, but was absent as the cells elongated, suggesting that nuclear Tos4 is lost in late S or G2. The duration of the presence of nuclear Tos4-GFP was about 60 min (Figure 1B) which was about 18% of the time of cell cycle (Figure 1C). Timing of nuclear Tos4-GFP relative to spindle duplication varied little between individual cells. We next examined the use of Tos4 as a dynamic marker to characterize S phase.

### Tos4 accumulation in cell cycle arrest defines early S phase

**Figure 2:**
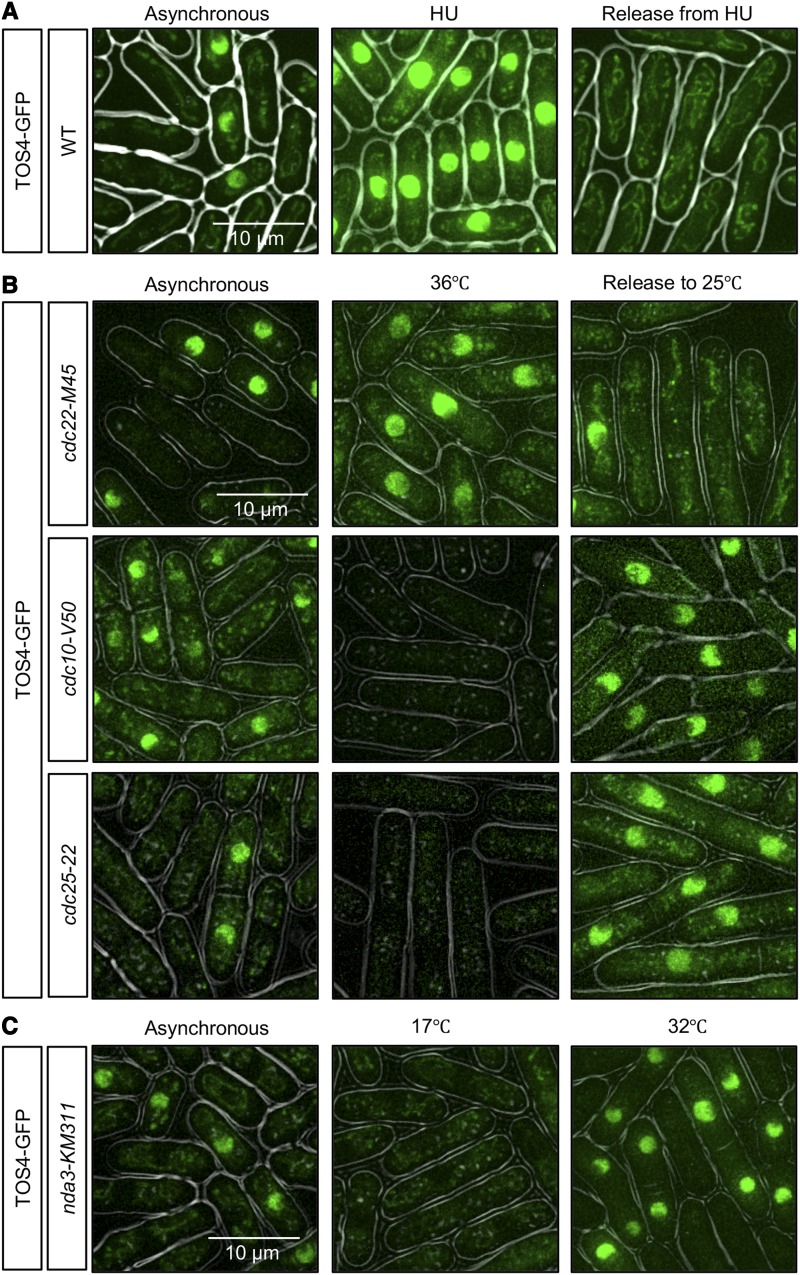
Tos4-GFP accumulate in the nuclei of cells arrested in early S phase. (A) WT cells were imaged for Tos4-GFP in asynchronous culture, after treatment with 12 mM HU for 4 h, and 1 h after release from HU. (B) Temperature-sensitive cell cycle mutants, *cdc22-M45* (S phase arrest), *cdc10-V50* (G1 phase arrest), *cdc25-22* (G2 phase arrest) were imaged for Tos4-GFP in asynchronous culture at 25°C, after 4 h at 36°C, or after 1 h-1.5 h after release to 25°C. (C) Cold-sensitive *nda3-KM311* (M phase arrest) was imaged for Tos4-GFP in asynchronous culture at 32°C, after 4 h at 17°C, or after 0.5 h after release to 32°C.

Treatment with hydroxyurea (HU) or temperature sensitive mutation of the ribonucleotide reductase component *cdc22* leads to depletion of nucleotide pools, and arrest of cells in early S phase, with a largely unreplicated DNA content (Timson 1975; Sarabia *et al.* 1993). We observed Tos4-GFP accumulation both in HU-treated or temperature-sensitive *cdc22-M45* at the restrictive temperature (Figure 2A,B). Tos4 was depleted when *cdc22-M45* cells were released back to the permissive temperature (25°), consistent with return to the cell cycle (Figure 2B). Cells arrested in G1 by mutation of the MBF transcription factor that regulates *tos4*+ expression (*cdc10-V50*) or in G2 by the mitotic inducing phosphatase (*cdc25-22*) showed no nuclear accumulation of Tos4 at 36° but gained nuclear Tos4 upon release to 25° (Figure 2B). Similarly, cells arrested at mitosis (*nda3-KM311*) had no nuclear Tos4 at 17° but gained nuclear Tos4 when released to 32° (Figure 2C).

**Figure 3:**
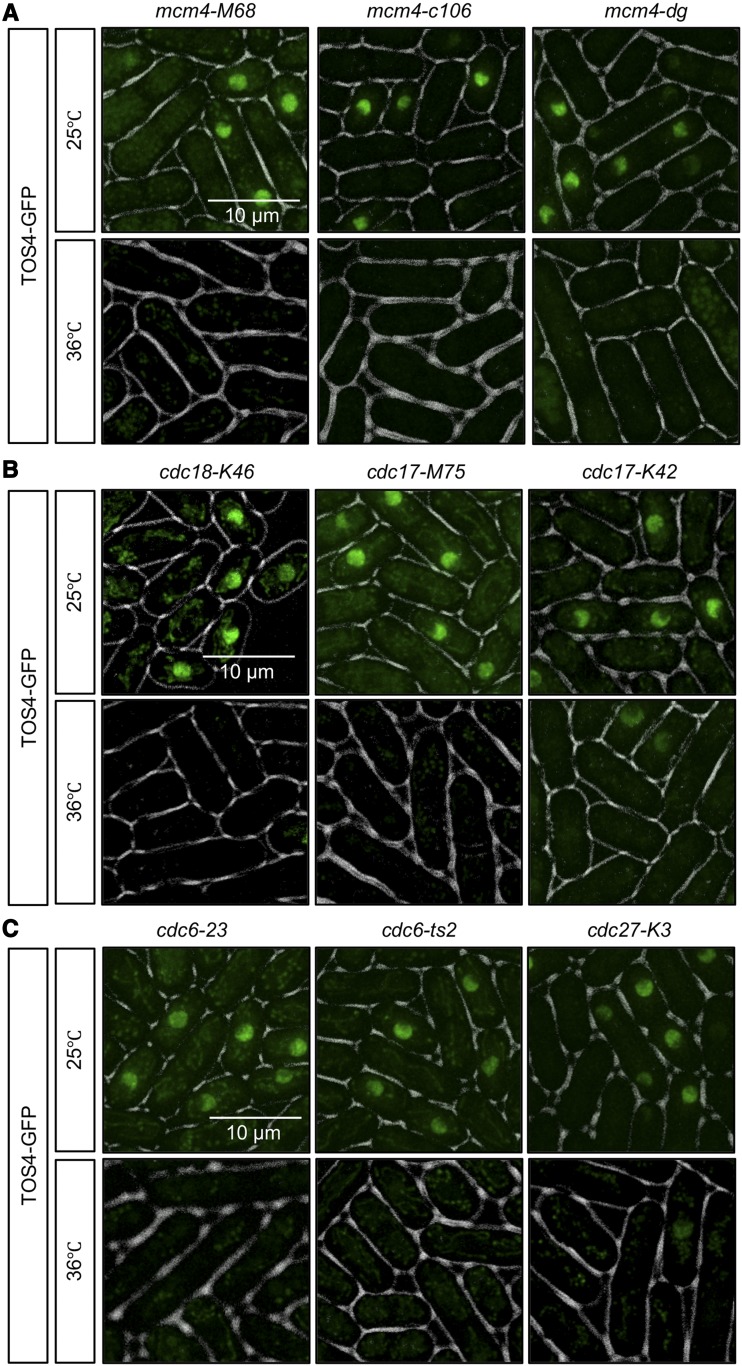
Replication mutants lack nuclear Tos4-GFP. Temperature-sensitive Mcm4 helicase mutants (*mcm4-M68*, *mcm4-c106*, *mcm4-dg*) (A), MCM loader mutant (*cdc18-K46*), ligase mutants (*cdc17-M45 and cdc17-K42*) (B), and polymerase delta mutants (*cdc6-23*, *cdc6-ts2*, *and cdc27-K3*) (C) were imaged for Tos4-GFP in asynchronous culture at 25°C or after 4 h at 36°C.

Next, we examined Tos4 accumulation in a variety of S phase mutants. During replication, the MCM helicase, which comprises six subunits, unwinds the DNA duplex and promote replication initiation and progression (Forsburg 2004). The canonical temperature-sensitive mutant *mcm4-M68 (mcm4-ts)* synthesizes a near 2C DNA content at restrictive temperature (36°) but shows low viability when released to permissive temperature (25°) (Nasmyth and Nurse 1981; Coxon *et al.* 1992; Liang *et al.* 1999; Sabatinos *et al.* 2015). A large C-terminal truncation mutant *mcm4-c106*, also shows 2C DNA content at 36° but much higher viability upon release than *mcm4-M68* (Nitani *et al.* 2008; Ranatunga and Forsburg 2016). A different temperature allele *mcm4-dg* that has a degron cassette added to *mcm4-M68*, undergoes rapid protein turnover at 36° with limited DNA synthesis and a 1C DNA content (Lindner *et al.* 2002; Sabatinos *et al.* 2015) although it fails to arrest divisions (Sabatinos *et al.* 2015). Interestingly, all three *mcm4* mutants lacked nuclear Tos4 when placed at 36°, even though they have different DNA contents and phenotypes (Figure 3A). We also tested temperature-sensitive mutants affecting the MCM loader *cdc18-K46*, DNA ligase mutants (*cdc17-M45* and *cdc17-K42*), and mutants affecting DNA polymerase delta subunits (*cdc6-23*, *cdc6-ts2*, and *cdc27-K3*). All of these arrest with a near 2C DNA content (Nasmyth and Nurse 1981). None maintained nuclear Tos4 (Figure 3B,C). Thus, Tos4 accumulation is different in early S phase (HU, *cdc22*) compared to late S phase mutants, and its accumulation is not limited by DNA content.

**Figure 4:**
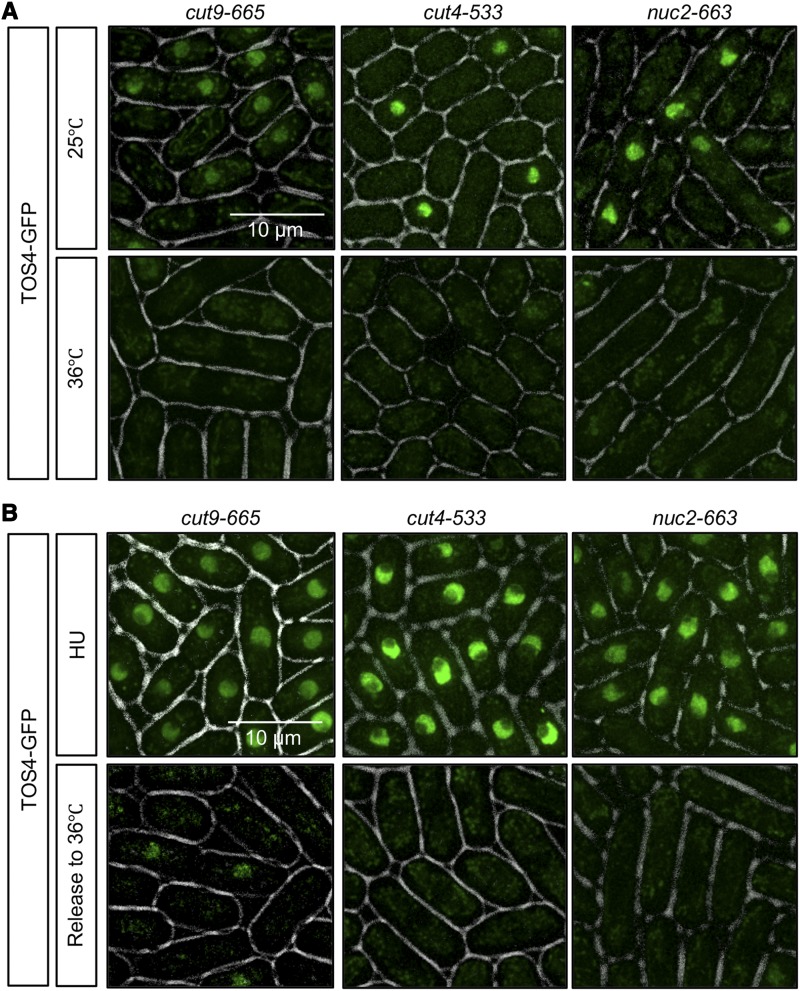
Tos4-GFP is not targeted for APC-mediated degradation. (A) Temperature-sensitive APC mutants (*cut9-665*, *cut4-533*, and *nuc2-663*) were imaged for Tos4-GFP in asynchronous culture at 25°C or after 4 h at 36°C. (B) APC mutants were imaged for Tos4-GFP after treatment with 12 mM HU for 4 h and 1 h after release from HU.

The Anaphase-Promoting Complex (APC) is an ubiquitin ligase that targets various proteins for proteasome-mediated degradation (Harper *et al.* 2002; Sivakumar and Gorbsky 2015). APC mutants have mitotic arrest with mostly 2C DNA content (Weinert and Hartwell 1993; Yuan *et al.* 2014). In budding yeast, Tos4 interacts with Cdh1, a WD40-repeat-containing activator of APC complex that recognizes degradation motifs in substrates (Ostapenko *et al.* 2012). Cdh1^Sc^ deletion results in partial stabilization of Tos4^Sc^ (Ostapenko *et al.* 2012) but not the temperature sensitive APC mutant *cdc23-1*^Sc^, suggesting Tos4^Sc^ protein turnover depends on multiple pathways. Fission yeast Tos4 does have potential APC recognition motifs (destruction box and KEN box) although the prediction score is not as high as Tos4 in budding yeast (Liu *et al.* 2012). We observed Tos4-GFP in three different temperature sensitive APC mutants: *cut9-665*, *cut4-533*, and *nuc2-663*. At 36°, Tos4 did not accumulate in any of these APC mutants (Figure 4A), consistent with a cell cycle arrest in mitosis. We pretreated APC mutants with HU and then released to 36°. If Tos4 protein is a target for APC-mediated degradation, we reasoned Tos4 would remain nuclear. HU-treated APC mutant cells accumulated nuclear Tos4 at both 25° and 36° but lost the signal when released from HU to 36° (Figure 4B and Figure S1A). Moreover, Ste9 (homologous to Cdh1^Sc^) deletion did not result in Tos4 accumulation (Figure S1B). Together these findings suggest that Tos4 is unlikely to be an APC target in fission yeast.

### Tos4 localization correlated with protein levels

**Figure 5:**
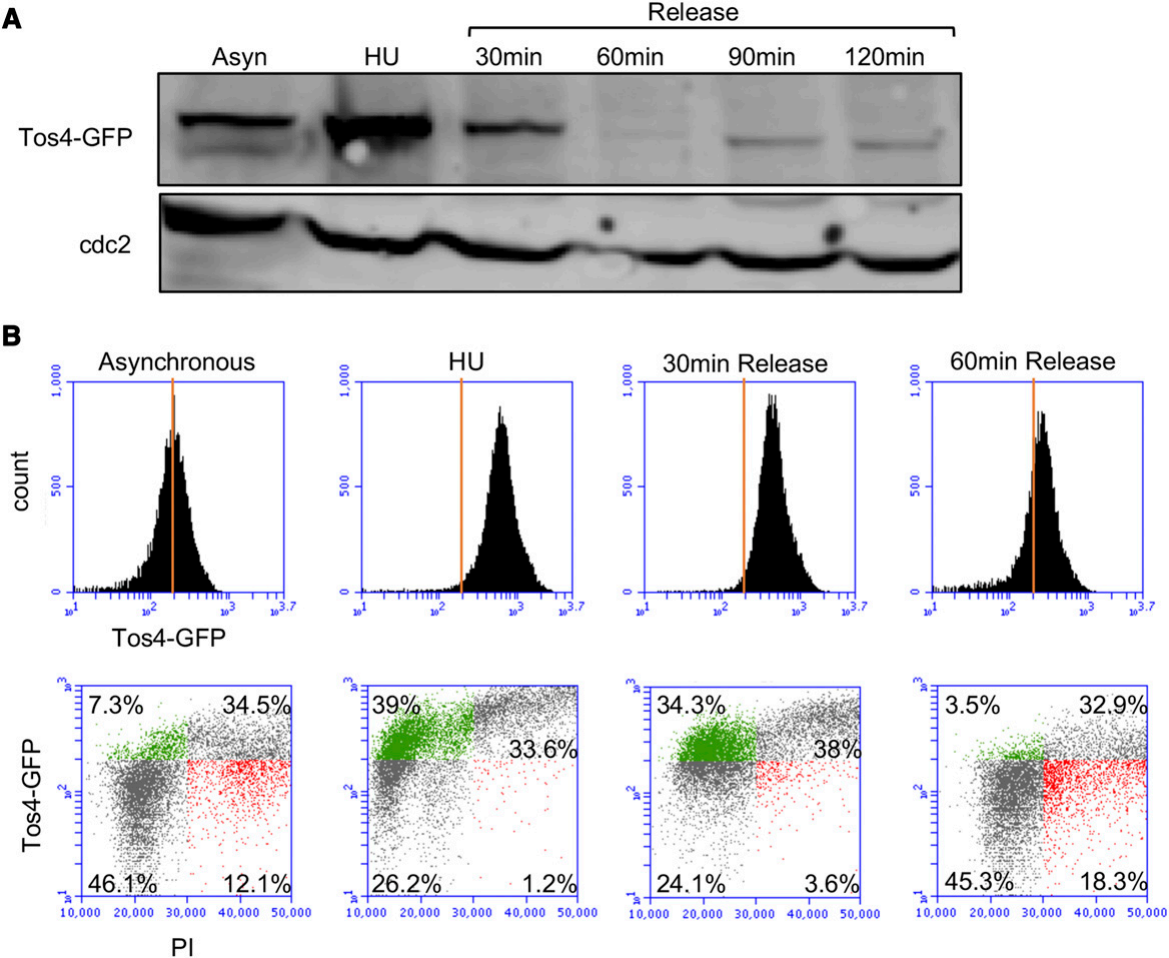
Tos4 protein level is increased in S phase cells arrested by HU. (A) WT cells with Tos4-GFP in asynchronous culture, after treatment with 12 mM HU, or after release from HU were lysed and immunoblotted for GFP and cdc2 (loading control). (B) WT cells used in (A) were fixed in 70% ethanol, and FACS analyzed for GFP and propidium iodide (PI). Green in scatter plot represents population with high GFP and low PI while red represents population with low GFP and high PI.

We determined whether observed accumulation of nuclear Tos4 is due to nuclear localization of Tos4 alone or whether it correlates with protein levels changes during S phase, using western blot analysis and flow cytometry (FACS) analysis. Lysates were collected from cells arrested in S phase with HU and released. Tos4 protein level increased in cells arrested in S phase compared to cells in asynchronous culture (Figure 5A). Tos4 protein level decreased to basal levels 30-60 min after release from HU. We also used FACS analysis to detect the GFP signal. This showed similar results, with the GFP peak increased in cells arrested in S phase and decreased back as cells were released from HU (Figure 5B). These results demonstrate that both Tos4 localization and protein turnover are regulated during S phase.

### Tos4 accumulation in early S phase is Cds1-dependent

**Figure 6:**
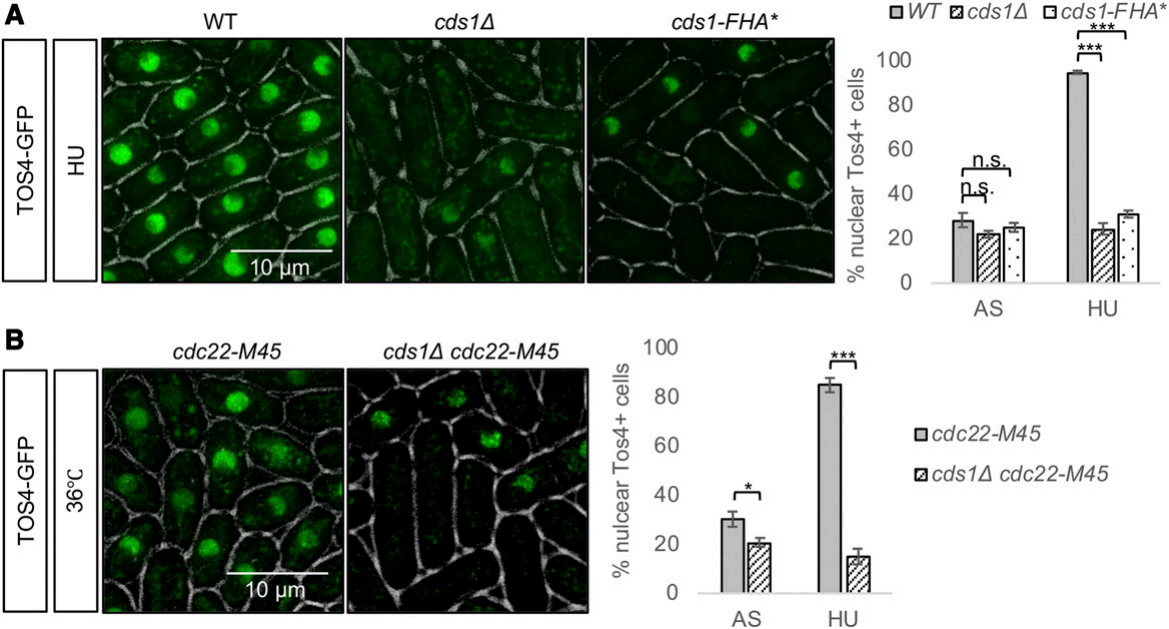
Cds1 is required for nuclear Tos4 accumulation in cells arrested in early S phase by HU or *cdc22-M45*. (A) *WT*, *cds1Δ*, cds1-FHA* cells were imaged for Tos4-GFP after treatment with 12 mM HU. Right, quantification of % cells with nuclear Tos4-GFP. (B) *cdc22-M45* and *cds1Δ cdc22-M45* cells were imaged for Tos4-GFP after 4 h at 36°C. Right, quantification of % cells with nuclear Tos4-GFP. N> 300 cells analyzed for each strain. A two-tailed Student’s *t*-test was used to determine significance: * *P* < 0.05, *** *P* < 0.001, n.s. not significant. Error bars represent Standard Error (SE).

HU blocks DNA synthesis by depleting deoxynucleoside triphosphate (dNTP) pools (Reichard 1988), which results in activation of the replication checkpoint kinase Cds1 (Lopes *et al.* 2001; Kai *et al.* 2005). Cds1 stabilizes replication forks and prevents cell division during replication arrest (Lindsay *et al.* 1998; Kai and Wang 2003). Previously, we showed that *cds1*∆ mutants fail to stop DNA synthesis during HU treatment, with lethal consequences (Sabatinos *et al.* 2012). Activation of Cds1 has been shown to upregulate the MBF transcription factor (de Bruin *et al.* 2008; Dutta *et al.* 2008; Chu *et al.* 2009; Bastos de Oliveira *et al.* 2012) (reviewed in (Smolka *et al.* 2012; Bertoli *et al.* 2013)). Consistent with this, we observed that nuclear Tos4 accumulation during HU treatment or in *cdc22-M45* arrest is Cds1-dependent (Figure 6A,B). We also observed that this requires the forkhead-associated domain (FHA) of Cds1, a phospho-peptide-binding module that mediates association with proteins such as Mrc1 and Mus81 (Boddy *et al.* 2000; Tanaka and Russell 2004). The cds1-fha* allele has mutations at two highly conserved residues (S79A and H82A) in the FHA domain and decreases DNA damage tolerance (Boddy *et al.* 2000). Similar to *cds1*Δ, the cds1-fha* cells treated with HU did not show nuclear Tos4 (Figure 6A). Kinase activity of Cds1 is also required for nuclear retention of Tos4 as kinase-dead Cds1 expression in *cds1Δ* did not retain nuclear Tos4 in HU (Figure S1C).

**Figure 7:**
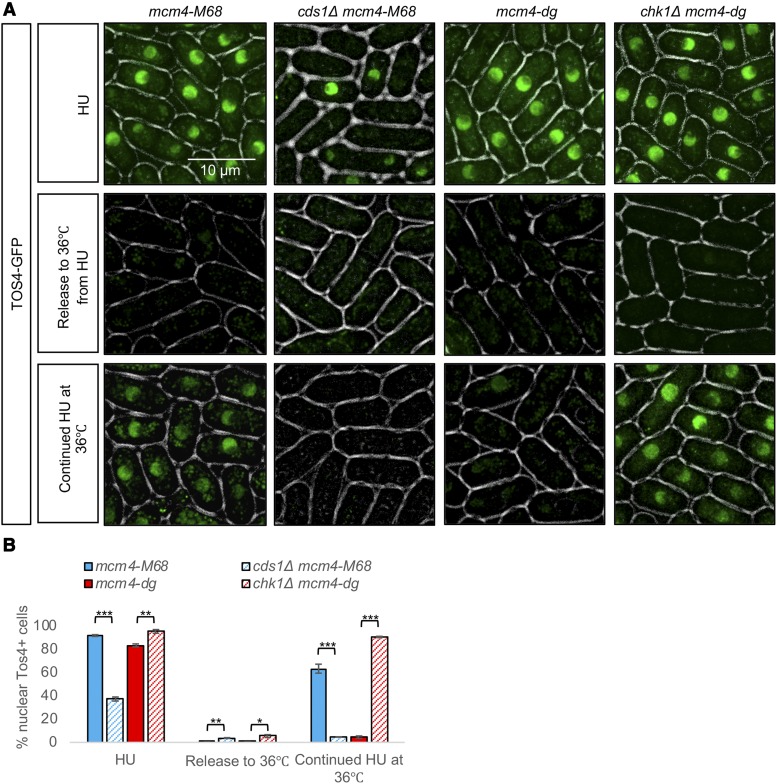
Continued Cds1 activation induces S phase arrest in *mcm4-M68* but not *mcm4-dg*. (A) *mcm4-M68*, *cds1Δ mcm4-M68*, *mcm4-dg*, *chk1Δ mcm4-dg* were imaged for Tos4-GFP after treatment with 12 mM HU at 25°C, after release from HU to 36°C, or after pre-treatment with HU at 25°C then transfer to 36°C. (B) Quantification of % cells with nuclear Tos4-GFP in (A). N> 300 cells analyzed for each strain. A two-tailed Student’s *t*-test was used to determine significance: * *P* < 0.05, ** *P* < 0.01, *** *P* < 0.001, Error bars represent Standard Error (SE).

We next examined whether activating Cds1 with HU first would be sufficient to maintain nuclear Tos4 in cells with temperature-sensitive mutations in replication mutants that normally do not accumulate Tos4. Temperature-sensitive *mcm4* mutants, *mcm4-M68* and *mcm4-dg*, treated with HU accumulated nuclear Tos4 as expected, but this was lost upon release from HU to 36° (Figure 7A,B), demonstrating that transient hyperactivation of Cds1 by HU is not sufficient to maintain nuclear Tos4. We next asked whether there was a difference if we maintained HU treatment at the restrictive temperature, so we shifted *mcm4-M68* and *mcm4-dg* from HU at 25° to HU at 36°. The *mcm4-M68* cells maintain nuclear Tos4 under both temperature conditions, and this depends upon Cds1 (Figure 7A,B). Other replication mutants *cdc45/sna41*, *cdc18*, *cdc6*, *cdc17*, and *cdc27* also maintain nuclear Tos4 in the continued presence of HU at 36° (Figure S2). Surprisingly, however, *mcm4-dg* cells do not maintain nuclear Tos4 in HU at 36° (Figure 7A,B). Additionally, we showed that this loss of Tos4 in HU at 36° in *mcm4-dg* is rescued by deletion of the damage checkpoint kinase Chk1 (Figure 7A,B).

**Figure 8:**
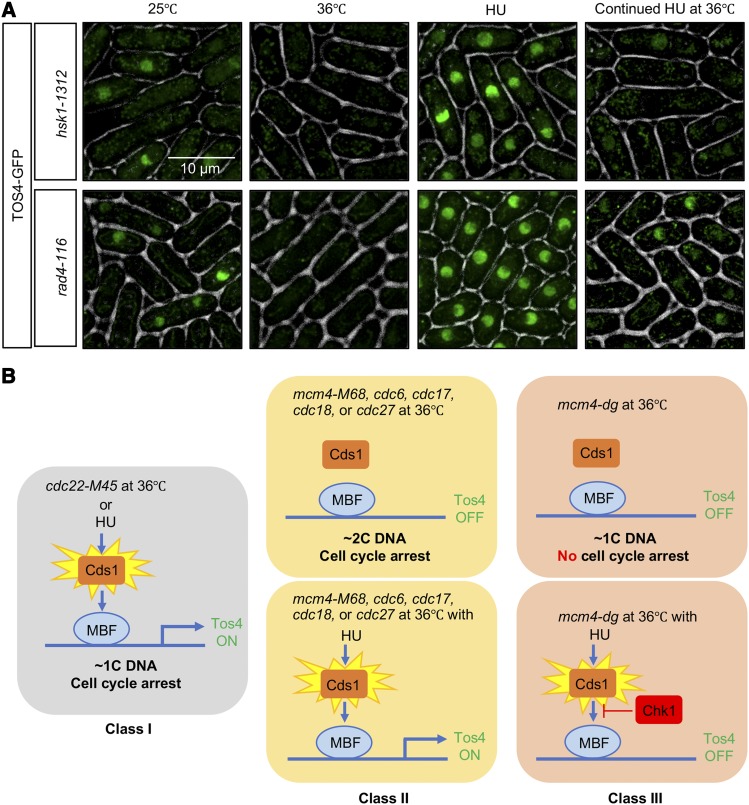
Replication mutants that bypass cell cycle arrest lack nuclear Tos4 despite continued Cds1 activation by HU. (A) Temperature-sensitive *hsk1-1312* and *rad4-116* cells were imaged for Tos4-GFP in asynchronous culture at 25°C, after 4h at 36°C, after treatment with 12 mM HU at 25°C, or after pre-treatment with HU at 25°C then transfer to 36°C. (B) Left, early replication stress (1C DNA) such as HU and *cdc22-M45* activates Cds1 and Tos4 expression is upregulated. Middle, replication mutants that complete most of DNA synthesis (2C DNA) fail to fully activate Cds1 and Tos4 expression is turned off unless in the continued presence of HU. Right, early replication mutant *mcm4-dg* (1C DNA) that enters mitosis fail to activate Cds1 and turn on Tos4 expression even in the presence of HU, in a Chk1-dependent manner.

The *mcm4-dg* allele is unusual as it bypasses normal cell cycle arrest and continues into mitosis despite the absence of substantial DNA synthesis (Sabatinos *et al.* 2015). We looked at two additional temperature sensitive replication mutants. The *hsk1*-1312 mutation affects, the catalytic subunit of the fission yeast Dbf4-dependent kinase (DDK) that regulates initiation of DNA replication via MCM, and *rad4-116 (cut5)* is also required for initiation, yet both proceed into mitosis at the restrictive temperature (Saka *et al.* 1994; McFarlane *et al.* 1997; Ostapenko *et al.* 2012). Similar to *mcm4-dg*, both these mutants lose nuclear Tos4 at 36° and fail to maintain nuclear Tos4 in continued presence of HU at 36° (Figure 8A).

## Discussion

Using an imaging-based approach, we demonstrate that nuclear Tos4 accumulation marks early S phase stage independent of DNA content, and allows us to identify three distinctive types of temperature sensitive S phase mutants (Figure 8B): Class 1 mutants arrest replication with a 1C DNA content that results in nuclear Tos4 accumulation; Class 2 are late S/G2 phase arrest mutants (2C DNA content) that lack nuclear Tos4 unless Cds1 is activated by the on-going presence of HU; Class 3 are early replication mutants (1C DNA) that fail to arrest the cell cycle and continue into mitosis, but lack nuclear Tos4 and cannot maintain Tos4 in the nucleus at the restrictive temperature even in the presence of HU.

These phenotypes may be partly distinguished by checkpoint activation. Tos4 is one of many genes whose expression is induced during G1-S transition and repressed in G2 in unperturbed cells, due to oscillation of the MBF transcription factor (Bastos de Oliveira *et al.* 2012). The activated replication checkpoint kinase Cds1 maintains high levels of G1-S transcription of these genes by preserving MBF activity (reviewed in (Bertoli *et al.* 2013)). Our data show that fission yeast requires active Cds1 to maintain Tos4 accumulation in cells arrested by HU-treatment or by *cdc22-M45* (Figure 2); in budding yeast, the Rad53 checkpoint kinase has a similar effect (Bastos de Oliveira *et al.* 2012). Lack of nuclear Tos4 accumulation in *cds1*-deleted cells is consistent with transcription and protein levels of Tos4 being similarly decreased in *S. pombe* corresponding to loss of MBF activity. Temperature-sensitive replication mutants that arrest with a nearly 2C DNA content (*mcm4-M68*, *cdc18-K46*, *cdc17-M75*, *cdc17-K42*, *cdc6-23*, *cdc6-ts2*, *cdc27-K3*) do not retain nuclear Tos4 at restrictive temperature (Figure 3), consistent with a failure to activate Cds1 (Lindsay *et al.* 1998). Conversely, at least some of these late mutants are known to activate Chk1 in response to double strand breaks, which is required for their arrest (Coxon *et al.* 1992; Forsburg and Nurse 1994; Liang *et al.* 1999; Bailis *et al.* 2008; Yin *et al.* 2008). The loss of Tos4 in these conditions is consistent with a previous study showing that Chk1 inhibits MBF (Ivanova *et al.* 2013). Thus, the two checkpoints have opposite effects on MBF. We conclude that the distinction in Tos4 accumulation in the arrested class 1 and class 2 mutants may represent which checkpoint is active, and the corresponding effect on Tos4 gene expression via MBF (upregulated by Cds1 and downregulated by Chk1).

The third class of mutants *mcm4-dg*, *hsk1-1312* and *rad4-116* have severe defects in DNA synthesis but nonetheless continue into mitosis without a cell cycle arrest, indicating that they have not activated either checkpoint, despite unreplicated DNA and evidence for DNA damage (Saka *et al.* 1997; Snaith *et al.* 2000; Lindner *et al.* 2002; Sabatinos *et al.* 2015). Similarly, they do not maintain nuclear Tos4 at the restrictive temperature, which we conclude may reflect their ongoing cell cycle progression. Consistent with this, we do not observe Tos4 accumulating in *chk1∆ mcm4-dg* at the restrictive temperature.

Interestingly, however, these class 3 mutants also fail to retain Tos4 if shifted to the restrictive temperature in the continued presence of HU, despite the absence of DNA synthesis (Figure 7A, 8A). Our previous data provide some insight into this difference. We showed that *mcm4-dg* cells at the restrictive temperature do not activate Chk1 (Sabatinos *et al.* 2015), and do not show evidence for double strand breaks as measure by accumulation of H2A(X) phosphorylation (Bailis *et al.* 2008). However, if we shift *mcm4-dg* mutants to 36° in the ongoing presence of HU, they do accumulate H2A(X) phosophorylation (Bailis *et al.* 2008), which suggests they have a different form of disruption or fork collapse than observed in the absence of HU. DSBs activate the Chk1 damage kinase, which we predict should repress the MBF and result in the loss of nuclearTos4. Consistent with this, we observe that a *chk1∆ mcm4-dg* double mutant maintains nuclear Tos4 in HU at the restrictive temperature. (We were unable to determine the effect in *hsk1-1312* and *rad4-116* double mutants because *chk1∆* has synthetic lethality or a growth defect in these backgrounds (Walworth *et al.* 1993; McFarlane *et al.* 1997; Snaith *et al.* 2000; Taricani and Wang 2006). Thus, we conclude that the failure to maintain Tos4 in HU at 36° for *mcm4-dg* reflects activation of Chk1, which is not the case in the absence of HU. Whether this accounts for the response of *rad4*, which is required for Chk1 activation (Furuya *et al.* 2004) remains to be determined.

Temperature-sensitive APC mutants (*cut9-665*, *cut4-533*, and *nuc2-663)* did not stabilize nuclear Tos4 when released from HU to the restrictive temperature, suggesting Tos4 may not be an APC target in fission yeast. Ste9, a WD-repeat protein homologous to budding yeast Cdh1, activates APC and promotes degradation of mitotic cyclins (Kitamura *et al.* 1998; Blanco *et al.* 2000). Cds1 phosphorylates and inhibits Ste9 to protect the MBF activator Rep2 from degradation (Chu *et al.* 2009), but we see no effect of *ste9∆* on Tos4 localization.

The presence of Tos4 in mutant backgrounds at restrictive conditions may be more precisely a measure for different pathways of checkpoint activation rather than position within S phase. Importantly, cells apparently blocked nominally in late S phase may actually be in G2 phase, consistent with recent evidence (Kelly and Callegari 2019). This raises the possibility that the distinction between S phase and G2 actually depends upon the ability to activate the damage checkpoint. Tos4 is known to be a phosphoprotein (Swaffer *et al.*, 2018), so it is possible it is directly regulated by check point kinases. Using various fluorescently-tagged cell cycle dependent proteins in combination with other cell cycle and checkpoint mutants will help elucidate whether or not there are distinctive changes that distinguish late S phase and G2 phase.

## References

[bib1] Alber A. B., Suter D. M., Alber A. B., and Suter D. M., 2019 Dynamics of protein synthesis and degradation through the cell cycle. Cell Cycle 18: 784–794. 10.1080/15384101.2019.1598725 30907235 PMC6527273

[bib2] Bailis J. M., Luche D. D., Hunter T., and Forsburg S. L., 2008 Minichromosome Maintenance Proteins Interact with Checkpoint and Recombination Proteins To Promote S-Phase Genome Stability. Mol. Cell. Biol. 28: 1724–1738. 10.1128/MCB.01717-07 18180284 PMC2258774

[bib3] Bajar B. T., Lam A. J., Badiee R. K., Oh Y., Chu J., 2016 Fluorescent indicators for simultaneous reporting of all four cell cycle phases. Nat. Methods 13: 993–996. 10.1038/nmeth.4045 27798610 PMC5548384

[bib4] Le Beau M. M., Rassool F. V., Neilly M. E., Iii R. E., Glover T. W., 1998 Replication of a common fragile site, FRA3B, occurs late in S phase and is delayed further upon induction: implications for the mechanism of fragile site induction. Hum. Mol. Genet. 7: 755–761. 10.1093/hmg/7.4.755 9499431

[bib5] Bergoglio V., Boyer A., Walsh E., Naim V., Legube G., 2013 DNA synthesis by Pol η promotes fragile site stability by preventing under-replicated DNA in mitosis. J. Cell Biol. 201: 395–408. 10.1083/jcb.201207066 23609533 PMC3639397

[bib48] Bastos de Oliveira F. M. B., Harris M. R., Brazauskas P., and De Bruin R. A. M., 2012 Linking DNA replication checkpoint to MBF cell-cycle transcription reveals a distinct class of G1/S genes. EMBO J. 31: 1798–1810. 10.1038/emboj.2012.27 22333912 PMC3321206

[bib6] Bertoli C., Skotheim J. M., and de Bruin R. A. M., 2013 Control of cell cycle transcription during G1 and S phases. Nat. Publ. Gr. 14: 518–528. 10.1038/nrm3629 PMC4569015 23877564

[bib7] Blanco M. A., Sanchez-Diaz A., de Prada J. M., and Moreno S., 2000 APC (ste9/srw1) promotes degradation of mitotic cyclins in G1 and is inhibited by cdc2 phosphorylation. EMBO J. 19: 3945–3955. 10.1093/emboj/19.15.3945 10921876 PMC306614

[bib8] Boddy M. N., Lopez-Girona A., Shanahan P., Interthal H., Heyer W. D., 2000 Damage tolerance protein Mus81 associates with the FHA1 domain of checkpoint kinase Cds1. Mol. Cell. Biol. 20: 8758–8766. 10.1128/MCB.20.23.8758-8766.2000 11073977 PMC86503

[bib10] Chu Z., Eshaghi M., Poon S. Y., and Liu J., 2009 A Cds1-Mediated Checkpoint Protects the MBF Activator Rep2 from Ubiquitination by Anaphase-Promoting Complex/Cyclosome – Ste9 at S-Phase Arrest in Fission Yeast. Mol. Cell. Biol. 29: 4959–4970. 10.1128/MCB.00562-09 19596787 PMC2738293

[bib11] Coxon A., Maundrell K., and Kearsey S. E., 1992 Fission yeast cdc2l + belongs to a family of proteins involved in an early step of chromosome replication. Nucleic Acids Res. 20: 5571–5577. 10.1093/nar/20.21.5571 1454522 PMC334388

[bib9] de Bruin R. A. M., Kalashnikova T. I., Aslanian A., Wohlschlegel J., Chahwan C., 2008 DNA replication checkpoint promotes G_1_-S transcription by inactivating the MBF repressor Nrm1. Proc. Natl. Acad. Sci. USA 105: 11230–11235. 10.1073/pnas.0801106105 18682565 PMC2516226

[bib12] Dutta C., Patel P. K., Rosebrock A., Oliva A., Leatherwood J., 2008 The DNA Replication Checkpoint Directly Regulates MBF-Dependent. Mol. Cell. Biol. 28: 5977–5985. 10.1128/MCB.00596-08 18662996 PMC2547018

[bib13] Escorcia W., and Forsburg S. L., 2017 Destabilization of the replication fork protection complex disrupts meiotic chromosome segregation. Mol. Biol. Cell 28: 2978–2997. 10.1091/mbc.e17-02-0101 28855376 PMC5662257

[bib14] Escorcia W., Shen K.-F., Yuan J., and Forsburg S. L., 2019 Examination of Mitotic and Meiotic Fission Yeast Nuclear Dynamics by Fluorescence Live-Cell Microscopy. J. Vis. Exp. 148: e59822 10.3791/59822 PMC6701690 31282894

[bib15] Forsburg S. L., 2004 Eukaryotic MCM Proteins : Beyond Replication Initiation. Microbiol. Mol. Biol. Rev. 68: 109–131. 10.1128/MMBR.68.1.109-131.2004 15007098 PMC362110

[bib16] Forsburg S. L., and Nurse P., 1994 The fission yeast cdc19+ gene encodes a member of the MCM family of replication proteins. J. Cell Sci. 107: 2779–2788. 7876346 10.1242/jcs.107.10.2779

[bib17] Furuya K., Poitelea M., Guo L., Caspari T., and Carr A. M., 2004 Chk1 activation requires Rad9 S/TQ-site phosphorylation to promote association with C-terminal BRCT domains of Rad4 TOPBP1. Genes Dev. 18: 1154–1164. 10.1101/gad.291104 15155581 PMC415640

[bib18] Gomez E. B., and Forsburg S. L., 2004 Analysis of the Fission Yeast Schizosaccharomyces pombe Cell Cycle, edited by Lieberman H. B. Humana Press, Totowa, NJ 10.1385/1-59259-646-0:93 14970648

[bib19] Green M. D., Sabatinos S. A., and Forsburg S. L., 2015 Microscopy Techniques to Examine DNA Replication in Fission Yeast, edited by Vengrova S., and Dalgaard J. Humana Press, New York 10.1007/978-1-4939-2596-4_2

[bib20] Harper J. W., Burton J. L., and Solomon M. J., 2002 The anaphase-promoting complex : it’s not just for mitosis any more. Genes Dev. 16: 2179–2206. 10.1101/gad.1013102 12208841

[bib77] Hayles J., Wood V., Jeffery L., Hoe K., Kim D., 2013 A genome-wide resource of cell cycle and cell shape genes of fission yeast. Open Biol. 3: 130053 10.1098/rsob.130053 23697806 PMC3866870

[bib21] Hodson J. A., Bailis J. M., and Forsburg S. L., 2003 Efficient labeling of fission yeast Schizosaccharomyces pombe with thymidine and BUdR. Nucleic Acids Res. 31: e134 10.1093/nar/gng134 14576334 PMC275491

[bib23] Ivanova T., Alves-Rodrigues I., Gomez-Escoda B., Dutta C., DeCaprio J. A., 2013 The DNA damage and the DNA replication checkpoints converge at the MBF transcription factor. Mol. Biol. Cell 24: 3350–3357. 10.1091/mbc.e13-05-0257 24006488 PMC3814153

[bib24] Kai M., Boddy M. N., Russell P., and Wang T. S. F., 2005 Replication checkpoint kinase Cds1 regulates Mus81 to preserve genome integrity during replication stress. Genes Dev. 19: 919–932. 10.1101/gad.1304305 15805465 PMC1080131

[bib25] Kai M., and Wang T. S. F., 2003 Checkpoint responses to replication stalling: Inducing tolerance and preventing mutagenesis. Mutat. Res. 532: 59–73. 10.1016/j.mrfmmm.2003.08.010 14643429

[bib26] Kelly T., and Callegari A. J., 2019 Dynamics of DNA replication in a eukaryotic cell. Proc. Natl. Acad. Sci. USA 116: 4973–4982. 10.1073/pnas.1818680116 30718387 PMC6421431

[bib27] Kiang L., Heichinger C., and Watt S., 2009 Cyclin-Dependent Kinase Inhibits Reinitiation of a Normal S-Phase Program during G2 in Fission Yeast. Mol. Cell. Biol. 29: 4025–4032. 10.1128/MCB.00185-09 19487461 PMC2715816

[bib28] Kiang L., Heichinger C., Watt S., Bähler J., and Nurse P., 2010 Specific replication origins promote DNA amplification in fission yeast. J. Cell Sci. 123: 3047–3051. 10.1242/jcs.067470 20736315 PMC2931602

[bib78] Kim D., Hayles J., Kim D., Wood V., Park H., 2010 Analysis of a genome-wide set of gene deletions in the fission yeast Schizosaccharomyces pombe. Nat. Biotechnol. 28: 617–623. 10.1038/nbt.1628 20473289 PMC3962850

[bib29] King M. C., and Drivas T. G., 2008 A Network of Nuclear Envelope Membrane Proteins Linking Centromeres to Microtubules. Cell 134: 427–438. 10.1016/j.cell.2008.06.022 18692466 PMC2617791

[bib30] Kitamura K., Maekawa H., and Shimoda C., 1998 Fission Yeast Ste9, a Homolog of Hct1/Cdh1 and Fizzy-related, Is a Novel Negative Regulator of Cell Cycle Progression during G1-Phase. Mol. Biol. Cell 9: 1065–1080. 10.1091/mbc.9.5.1065 9571240 PMC25330

[bib31] Lang G. I., and Murray A. W., 2011 Mutation Rates across Budding Yeast Chromosome VI Are Correlated with Replication Timing. Genome Biol. Evol. 3: 799–811. 10.1093/gbe/evr054 21666225 PMC3170098

[bib32] Liang D. T., Hodson J. A., and Forsburg S. L., 1999 Reduced dosage of a single fission yeast MCM protein causes genetic instability and S phase delay. J. Cell Sci. 112: 559–567. 9914167 10.1242/jcs.112.4.559

[bib33] Lindner K., Grega J., Montgomery S., and Kearsey S. E., 2002 Essential Role of MCM Proteins in Premeiotic DNA Replication. Mol. Biol. Cell 13: 435–444. 10.1091/mbc.01-11-0537 11854402 PMC65639

[bib34] Lindsay H. D., Griffiths D. J. F., Edwards R. J., Christensen P. U., Murray J. M., 1998 S-phase-specific activation of Cds1 kinase defines a subpathway of the checkpoint response in Schizosaccharomyces pombe. Genes Dev. 12: 382–395. 10.1101/gad.12.3.382 9450932 PMC316487

[bib35] Liu Z., Yuan F., Ren J., Cao J., Zhou Y., 2012 GPS-ARM : Computational Analysis of the APC / C Recognition Motif by Predicting D-Boxes and KEN-Boxes. PLoS One 7: e34370 10.1371/journal.pone.0034370 22479614 PMC3315528

[bib36] Lopes M., Cotta-Ramusino C., Pellicioli A., Liberi G., Plevani P., 2001 The DNA replication checkpoint response stabilizes stalled replication forks. Nature 412: 557–561. 10.1038/35087613 11484058

[bib37] MacNeill S., and Fantes P., 1997 Genetic and physiological analysis of DNA replication in fission yeast. Methods Enzymol. 283: 440–459. 10.1016/S0076-6879(97)83036-4 9251040

[bib38] Malumbres M., 2014 Cyclin-dependent kinases. Genome Biol. 15: 122 10.1186/gb4184 25180339 PMC4097832

[bib39] McFarlane R. J., Carr A. M., and Price C., 1997 Characterisation of the Schizosaccharomyces pombe rad4/cut5 mutant phenotypes: dissection of DNA replication and G2 checkpoint control function. Mol. Gen. Genet. 255: 332–340. 10.1007/s004380050504 9268024

[bib40] Minet M., Nurse P., Thuriaux P., and Mitchison J. M., 1979 Uncontrolled Septation in a Cell Division Cycle Mutant of the Fission Yeast Schizosaccharomyces pombe. J. Bacteriol. 137: 440–446. 762020 10.1128/jb.137.1.440-446.1979 PMC218468

[bib41] Minocherhomji S., Ying S., Bjerregaard V. A., Bursomanno S., Aleliunaite A., 2015 Replication stress activates DNA repair synthesis in mitosis. Nature 528: 286–290. 10.1038/nature16139 26633632

[bib42] Nasmyth K., and Nurse P., 1981 Cell Division Cycle Mutants Altered in DNA Replication and Mitosis in the Fission Yeast Schizosaccharomyces pombe. Mol. Gen. Genet. 182: 119–124. 10.1007/BF00422777 6943408

[bib43] Nasmyth K., Nurse P., and Fraser R., 1979 The Effect of Cell Mass on the Cell Cycle Timing and Duration of S-Phase in Fission Yeast. J. Cell Sci. 39: 215–233. 528581 10.1242/jcs.39.1.215

[bib45] Nitani N., Yadani C., Yabuuchi H., Masukata H., and Nakagawa T., 2008 Mcm4 C-terminal domain of MCM helicase prevents excessive formation of single-stranded DNA at stalled replication forks. Proc. Natl. Acad. Sci. USA 105: 12973–12978. 10.1073/pnas.0805307105 18753627 PMC2529087

[bib46] Nurse P., Thuriaux P., and Nasmyth K., 1976 Genetic Control of the Cell Division Cycle in the Fission Yeast Schizosaccharomyces pombe. Mol. Gen. Genet. 146: 167–178. 10.1007/BF00268085 958201

[bib47] Oki T., Nishimura K., Kitaura J., Togami K., Maehara A., 2014 A novel cell-cycle-indicator, mVenus-p27K 2, identifies quiescent cells and visualizes G0–G1 transition. Sci. Rep. 4: 4012 10.1038/srep04012 24500246 PMC3915272

[bib49] Ostapenko D., Burton J. L., and Solomon M. J., 2012 Identification of Anaphase Promoting Complex Substrates in S. cerevisiae. PLoS One 7: e45895 10.1371/journal.pone.0045895 23049888 PMC3458821

[bib50] Peng X., Karuturi R. K. M., Miller L. D., Lin K., Jia Y., 2005 Identification of Cell Cycle-regulated Genes in Fission Yeast. Mol. Biol. Cell 16: 1026–1042. 10.1091/mbc.e04-04-0299 15616197 PMC551471

[bib51] Piel M., and Tran P. T., 2009 Cell Shape and Cell Division in Fission Yeast. Curr. Biol. 19: R823–R827. 10.1016/j.cub.2009.08.012 19906584 PMC2997724

[bib52] Ranatunga N. S., and Forsburg S. L., 2016 Characterization of a Novel MMS-Sensitive Allele of Schizosaccharomyces pombe mcm4 +. G3 (Bethesda) 6: 3049–3063. 10.1534/g3.116.033571 27473316 PMC5068930

[bib53] Reichard P., 1988 Interactions between deoxyribonucleotide and DNA synthesis. Annu. Rev. Biochem. 57: 349–374. 10.1146/annurev.bi.57.070188.002025 3052277

[bib54] Sabatinos S. A., and Forsburg S. L., 2015 a Managing Single-Stranded DNA during Replication Stress in Fission Yeast. Biomolecules 5: 2123–2139. 10.3390/biom5032123 26393661 PMC4598791

[bib55] Sabatinos S. A., and Forsburg S. L., 2015 b Measuring DNA Content by Flow Cytometry in Fission Yeast, edited by Vengrova S., and Dalgaard J. Humana Press, New York 10.1007/978-1-4939-2596-4_5

[bib56] Sabatinos S. A., Green M. D., and Forsburg S. L., 2012 Continued DNA Synthesis in Replication Checkpoint Mutants Leads to Fork Collapse. Mol. Cell. Biol. 32: 4986–4997. 10.1128/MCB.01060-12 23045396 PMC3510540

[bib57] Sabatinos S. A., Ranatunga N. S., Yuan J., Green M. D., Forsburg S. L., 2015 Replication stress in early S phase generates apparent micronuclei and chromosome rearrangement in fission yeast. Mol. Biol. Cell 26: 3439–3450. 10.1091/mbc.E15-05-0318 26246602 PMC4591689

[bib58] Saka Y., Esashi F., Matsusaka T., Mochida S., and Yanagida M., 1997 Damage and replication checkpoint control in fission yeast is ensured by interactions of Crb2, a protein with BRCT motif, with Cut5 and Chk1. Genes Dev. 11: 3387–3400. 10.1101/gad.11.24.3387 9407031 PMC316798

[bib59] Saka Y., Fantes P., Sutani T., Mclnerny C., Creanor J., 1994 Fission yeast cut5 links nuclear chromatin and M phase regulator in the replication checkpoint control. EMBO J. 13: 5319–5329. 10.1002/j.1460-2075.1994.tb06866.x 7957098 PMC395488

[bib60] Sakaue-Sawano A., Kurokawa H., Morimura T., Hanyu A., Hama H., 2008 Visualizing Spatiotemporal Dynamics of Multicellular Cell-Cycle Progression. Cell 132: 487–498. 10.1016/j.cell.2007.12.033 18267078

[bib61] Sarabia M. F., Mcinerny C., Harris P., Gordon C., and Fantes P., 1993 The cell cycle genes cdc22+ and suc22+ of the fission yeast Schizosaccharomyces pombe encode the large and small subunits of ribonucleotide reductase. Mol. Gen. Genet. 238: 241–251. 8479429 10.1007/BF00279553

[bib62] Sawin K. E., and Tran P. T., 2006 Cytoplasmic microtubule organization in fission yeast. Yeast 23: 1001–1014. 10.1002/yea.1404 17072892 PMC5942541

[bib63] Schindelin J., Arganda-carreras I., Frise E., Kaynig V., Longair M., 2012 Fiji : an open-source platform for biological-image analysis. Nat. Methods 9: 676–682. 10.1038/nmeth.2019 22743772 PMC3855844

[bib64] Shen K.-F., and Forsburg S. L., 2019 Overlapping Roles in Chromosome Segregation for Heterochromatin Protein 1 (Swi6) and DDK in Schizosaccharomyces pombe. Genetics 212: 417–430. 10.1534/genetics.119.302125 31000521 PMC6553818

[bib65] Sivakumar S., and Gorbsky G. J., 2015 Spatiotemporal regulation of the anaphase-promoting complex in mitosis. Nat. Publ. Gr. 16: 82–94. 10.1038/nrm3934 PMC4386896 25604195

[bib66] Smolka M. B., De Oliveira F. M. B., Harris M. R., Smolka M. B., De Oliveira F. M. B., 2012 The checkpoint transcriptional response : Make sure to turn it off once you are satisfied. Cell Cycle 11: 3166–3174. 10.4161/cc.21197 22895177 PMC3466515

[bib67] Snaith H. A., Brown G. W., and Forsburg S. L., 2000 Schizosaccharomyces pombe Hsk1p Is a Potential Cds1p Target Required for Genome Integrity. Mol. Cell. Biol. 20: 7922–7932. 10.1128/MCB.20.21.7922-7932.2000 11027263 PMC86403

[bib68] Stamatoyannopoulos J. A., Adzhubei I., Thurman R. E., Kryukov G. V., and Mirkin S. M., 2009 Human mutation rate associated with DNA replication timing. Nat. Genet. 41: 393–395. 10.1038/ng.363 19287383 PMC2914101

[bib79] Swaffer M. P., Jones A. W., Flynn H. R., Snijders A. P., and Nurse P., 2018 Quantitative Phosphoproteomics Reveals the Signaling Dynamics of Cell-Cycle Kinases in the Fission Yeast Schizosaccharomyces pombe. Cell Reports 24: 503–514. 10.1016/j.celrep.2018.06.036 29996109 PMC6057490

[bib80] Swaffer M. P., Jones A. W., Flynn H. R., Snijders A. P., Swaffer M. P., 2016 CDK Substrate Phosphorylation and Ordering the Cell Cycle. Cell 167: 1750–1750.e16 10.1016/j.cell.2016.11.034 27984725 PMC5161751

[bib69] Tanaka K., and Russell P., 2004 Cds1 Phosphorylation by Rad3-Rad26 Kinase Is Mediated by Forkhead-associated Domain Interaction with Mrc1 *. J. Biol. Chem. 279: 32079–32086. 10.1074/jbc.M404834200 15173168

[bib70] Taricani L., and Wang T. S. F., 2006 Rad4 TopBP1, a Scaffold Protein, Plays Separate Roles in DNA Damage and Replication Checkpoints and DNA Replication. Mol. Biol. Cell 17: 3456–3468. 10.1091/mbc.e06-01-0056 16723501 PMC1525248

[bib71] Timson J., 1975 Hydroxyurea. Mutat. Res. 32: 115–131. 10.1016/0165-1110(75)90002-0 765790

[bib81] Vachon L., Wood J., Kwon E.-J. G., Laderoute A., Chatfield-Reed K., 2013 Functional Characterization of Fission Yeast Transcription Factors by Overexpression Analysis. Genetics 194: 873–884. 10.1534/genetics.113.150870 23695302 PMC3730917

[bib72] Walworth N., Davey S., and Beach D., 1993 Fission yeast chk1 protein kinase links the rad checkpoint pathway to cdc2. Nature 363: 368–371. 10.1038/363368a0 8497322

[bib73] Weinert T. A., and Hartwell L. H., 1993 Cell Cycle Arrest of cdc Mutants and Specificity of the RAD9 Checkpoint. Genet. Soc. Am. 134: 63–80. 10.1093/genetics/134.1.63 PMC1205445 8514150

[bib22] Widrow R. J., Hansen R. S., Kawame H., Gartler S. M., and Laird C. D., 1998 Very late DNA replication in the human cell cycle. Proc. Natl. Acad. Sci. USA 95: 11246–11250. 10.1073/pnas.95.19.11246 9736721 PMC21627

[bib74] Yin L., Locovei A. M., and D’Urso G., 2008 Activation of the DNA Damage Checkpoint in Mutants Defective in DNA Replication Initiation. Mol. Biol. Cell 19: 4374–4382. 10.1091/mbc.e08-01-0020 18667534 PMC2555949

[bib75] Yuan X., Srividhya J., De Luca T., Lee J. E., Pomerening J. R., 2014 Uncovering the role of APC-Cdh1 in generating the dynamics of S-phase onset. Mol. B 25: 441–456. 10.1091/mbc.E13-08-0480 PMC3923637 24356446

[bib44] Zhang B., Bueno A., Hickson I. D., and Chu W. K., 2019 DNA replication stress and its impact on chromosome segregation and tumorigenesis. Semin. Cancer Biol. 55: 61–69. 10.1016/j.semcancer.2018.04.005 29692334

[bib76] Zeman M. K., and Cimprich K. A., 2014 Causes and consequences of replication stress. Nat. Cell Biol. 16: 2–9. 10.1038/ncb2897 24366029 PMC4354890

